# Expanding the Phenotypic Spectrum of PRKD1 Gain-of-Function Syndrome: Congenital Heart Disease, Bilateral Carotid Dissections, and Ectodermal Dysplasia in an Adult Patient

**DOI:** 10.3390/genes17091048

**Published:** 2026-08-30

**Authors:** Pedram Kharaziha, Klara Junker, Josefine Åhsberg, Antheia Kissopoulou

**Affiliations:** 1 Clinical Department of Clinical Genetics, Region Östergötland, 58185 Linköping, Sweden; pedram.kharaziha@regionostergotland.se; 2 Department of Biomedical and Clinical Sciences, Linköping University, 58185 Linköping, Sweden; 3 Clinical Department of Precision Medicine Laboratory, Region Östergötland, 58185 Linköping, Sweden; klara.junker@regionostergotland.se (K.J.);; 4 Department of Internal Medicine, County Council of Jönköping, 58185 Linköping, Sweden; 5 Department of Health, Medicine and Caring Sciences, Linköping University, 58185 Linköping, Sweden

**Keywords:** artery, dissection, ectodermal dysplasia, arteriopathy, gain-of-function variant, vascular phenotype, CHDED syndrome

## Abstract

Background: *PRKD1*-related congenital heart defects and ectodermal dysplasia syndrome (CHDED) is a rare multisystem developmental disorder caused by heterozygous gain-of-function variants in *PRKD1*. Reported phenotypes include congenital heart defects, ectodermal abnormalities, limb anomalies, and neurodevelopmental impairment, while vascular manifestations remain poorly characterized. We report an adult patient with a de novo *PRKD1* variant and bilateral carotid artery dissections. Methods: Clinical, cardiovascular, neurological, and genetic evaluations were performed, including SNP-microarray and trio whole-exome sequencing with additional analysis of genes associated with heritable thoracic aortic and connective tissue disorders. Results: The patient was diagnosed at 35 years of age with a de novo *PRKD1* c.1808G>A p.(Arg603His) variant. He had childhood-onset congenital heart disease, ectodermal and skeletal abnormalities, hearing impairment, infertility, and learning difficulties. In adulthood, he developed bilateral carotid artery dissections and mild aortic dilatation. No additional pathogenic or likely pathogenic variants were identified in the evaluated connective tissue and heritable aortic disease genes. The *PRKD1* variant was absent or extremely rare in population databases and had supporting functional evidence for a gain-of-function effect. Conclusions: This case raises the possibility of a previously unrecognized vascular manifestation of *PRKD1*-related CHDED. Although a biological association is plausible, causality cannot be established from a single case. Further clinical and functional studies are needed to determine whether vascular fragility is a recurrent feature of *PRKD1*-related disease. This case also highlights the importance of considering rare genetic syndromes in patients with unexplained arteriopathy and congenital heart disease.

## 1. Introduction

Protein kinase D (PKD) is a family of three evolutionarily conserved serine/threonine kinases involved in multiple intracellular signaling pathways regulating cellular proliferation, differentiation, polarity, angiogenesis, apoptosis, and cardiac development [1,2,3,4,5,6,7,8]. The first member of the family, *PRKD1* (OMIM * 605435), was discovered in 1994 by Valverde and colleagues [9], followed by the identification of the other two family members, PRKD2 [10] and PRKD3 [11]. PKD1 is a 912-amino-acid protein encoded by the PRKD1 gene, which is located on the long arm of chromosome 14 at band 14q12. PRKD1 comprises 18 exons and is expressed across multiple tissues, including the heart, smooth muscle, lung, brain, kidney, pancreas, and prostate [12]. The effect of PKD proteins is mediated through several key signaling cascades, notably the RAS-MAPK-ERK, MEK-JNK, and PI3K/AKT pathways, as well as through modulation of autophagy [7].

Beyond its role in cancer biology, *PRKD1* has been implicated in a range of physiological and pathological processes. It contributes to the pathogenesis of hypertrophic cardiomyopathy and regulates vasodilation through signaling pathways involving AKT/mTOR, nitric oxide synthase (NOS), and nuclear factors of activated T cells (NFATs) [7]. Functional dysregulation of *PRKD1* has been implicated in cardiac morphogenesis, angiogenesis, endothelial signalling, inflammatory responses, vascular homeostasis, and cellular proliferation [7,13].

According to the Online Mendelian Inheritance in Man (OMIM) database, the Clinical Genome Resource (ClinGen) database, and The Human Gene Mutation Database (HGMD), *PRKD1* (OMIM * 605435) is currently the only member of the PKD family for which a definitive gene–disease relationship has been established. Heterozygous gain-of-function variants in *PRKD1* have recently been associated with congenital heart defects and ectodermal dysplasia syndrome (CHDED) [5,14], an exceptionally rare multisystem developmental disorder characterized by congenital cardiac malformations, ectodermal abnormalities, craniofacial dysmorphism, limb anomalies, and neurodevelopmental impairment. The roles of the other two family members, *PRKD2* (OMIM * 607074) and *PRKD3* (OMIM * 607077), are still under investigation.

**Figure 1:**
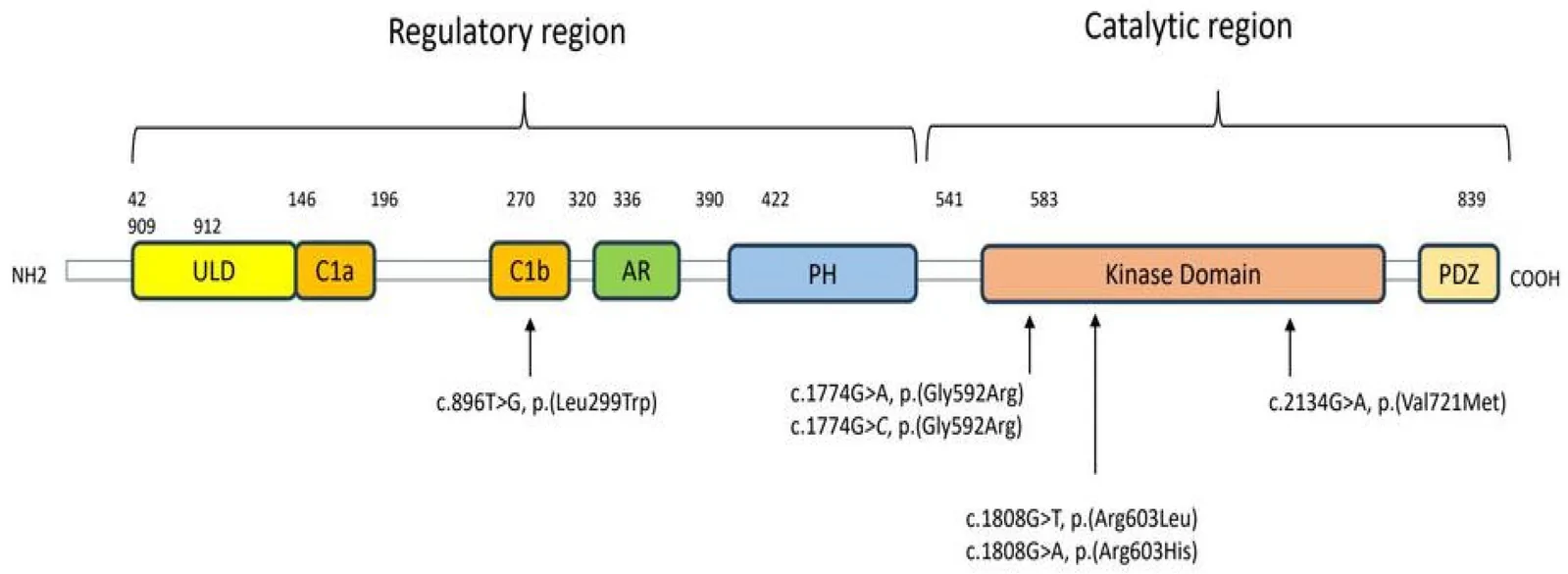
Domain structure of PKD1-protein: In N-terminal regulatory region, there is a ubiquitin-like domain (ULD) for dimerization, a C1 domain (C1a and C1b) that binds diacylglycerol, the acidic amino-acid-rich region (AR), and a pleckstrin homology (PH) domain for autoinhibition. The C-terminal catalytic region contains a kinase domain and a PDZ domain which facilitates protein substrate recognition.

The PKD1 protein consists of an N-terminal regulatory region and a C-terminal catalytic region (Figure 1) [7]. The regulatory region spans amino acids 42–541 and contains several important functional domains, including an alanine/proline-rich region, a ubiquitin-like domain (ULD) involved in dimerization during activation, and two diacylglycerol (DAG)-binding C1 domains (C1a and C1b). This region also contains a pleckstrin homology (PH) domain, which mediates autoinhibition of the protein. The C-terminal catalytic region extends from amino acid 542 to the end of the protein. It contains a catalytic kinase domain of approximately 256 amino acids, followed by a PDZ-binding motif that facilitates substrate recognition and protein–protein interactions. This overall structural organization is highly conserved among all three PKD family members.

PKD proteins can be activated by a variety of extracellular stimuli, including cellular stress, hormones, growth factors, and neuropeptides, through signaling pathways involving G-protein-coupled receptors (GPCRs) or receptor tyrosine kinases (RTKs). Upon activation, PKDs translocate from the plasma membrane to the nucleus, where they regulate the activity of several transcription factors.

### Role of PKD1 in Cardiomyocytes and Mouse Heart Development

PKD1, PKD2, and PKD3 are co-expressed in myocytes; however, several lines of evidence suggest that activation of these proteins occurs independently [15]. First, homozygous deletion of exon 2 in *PRKD1* in mouse cardiomyocytes causes a significant reduction in *PRKD1* mRNA levels without any compensatory increase in *PRKD2* or *PRKD3* transcript levels. Second, PKD1 is specifically activated by α1-adrenergic receptor (α1-AR) agonists, whereas PKD2 and PKD3 are activated by the protease-activated receptor agonist thrombin or the endothelial differentiation gene receptor agonist sphingosine-1-phosphate (S1P). As mentioned previously, the PKD family is involved in activation of RAS signaling, which also plays a well-established role in congenital heart disease (CHD).

Fielitz et al. demonstrated that activation of PKD1 is essential for mediating stress-dependent cardiac remodeling and transcriptional reprogramming in the murine heart [8]. They further reported that mice with cardiac-specific deletion of PKD1 exhibit an impaired response to stress stimuli compared to control mice expressing PKD1. This attenuated response is characterized by reduced cardiac hypertrophy, fibrosis, and activation of the fetal gene program.

Waheed-Ullah et al. analyzed a transgenic mouse model carrying a deletion of exon 2 in *PRKD1*, resulting in a loss-of-function allele [16]. They demonstrated that homozygous deletion of *PRKD1* is embryonically lethal and associated with a spectrum of cardiac phenotypes, ranging from isolated anomalies to complex forms of congenital heart disease. Five of six homozygous mutant mice exhibited CHD, including one case of a perimembranous ventricular septal defect (VSD), one dysplastic pulmonary valve, and one bicuspid aortic valve (BAV). Additionally, atrioventricular septal defects (AVSDs) were observed in two mice. In contrast, 97% of *PRKD1* heterozygous mice displayed normal cardiac development. Importantly, these findings reflect loss of PRKD1 function and do not directly model the heterozygous gain-of-function mechanism underlying CHDED. While loss-of-function studies demonstrate an essential role for PKD1 in cardiac development, gain-of-function variants may produce distinct effects through altered or increased downstream signaling, potentially influencing tissue morphogenesis and vascular homeostasis.

To date, only a limited number of individuals with *PRKD1*-related CHDED syndrome have been described worldwide [13,14,17,18]. Reported cardiovascular manifestations primarily include congenital structural heart disease, while vascular complications have not been well characterized. Experimental studies [14] suggest that *PRKD1* signaling participates in endothelial biology, angiogenesis, vascular remodeling, and inflammatory pathways, raising the possibility that dysregulation of this pathway could contribute to vascular fragility or arteriopathy.

Here, we describe an adult patient harboring a heterozygous, likely pathogenic gain-of-function PRKD1 variant, c.1808G>A p.(Arg603His), presenting with a complex multisystem phenotype including congenital heart disease, ectodermal abnormalities, infertility, and bilateral carotid artery dissections. This case substantially expands the known vascular and adult phenotype associated with PRKD1-related disease and raises the possibility that dysregulated PKD1 signaling, rather than reduced kinase activity, may contribute to vascular manifestations in affected individuals.

## 2. Material and Methods

### 2.1. Informed Consent

Written informed consent was obtained from the patient and his parents for the publication of clinical and molecular data and accompanying photographs.

### 2.2. SNP-Microarray Analysis:

Peripheral blood was collected in an EDTA tube, and germline DNA was extracted. The patient’s DNA was analyzed using a single-nucleotide polymorphism (SNP) microarray (CytoScan HD Array) CytoScan HD Array (Thermo Fisher Scientific, Waltham, MA, USA) and Chromosome Analysis Suite version 4.5 software Chromosome Analysis Suite (ChAS), version 4.5 (Thermo Fisher Scientific, Waltham, MA, USA). No copy number variants (CNVs) larger than 50 kb were identified that could explain the patient’s clinical phenotype.

### 2.3. Whole-Exome Sequencing

Whole-exome sequencing (WES) was carried out using the Twist Human Core Exome Plus RefSeq panel, (Twist Bioscience, South San Francisco, CA, USA which includes approximately 20,000 genes derived from the CCDS database and expanded with additional RefSeq content for enhanced clinical coverage. Library preparation was performed using the Twist Human Core Exome plus RefSeq panel and sequenced on an Illumina NextSeq 500 platform Illumina NextSeq 500 platform (Illumina, Inc., San Diego, CA, USA). following standard protocols. Sequence alignment and variant calling were conducted using DNAseq (Sentieon) DNAseq (Sentieon, Inc., San Jose, CA, USA with GRCh37 as the reference genome, and variants were annotated using Emedgene (Illumina). Genetic analysis included phenotype-driven filtering based on the following Human Phenotype Ontology (HPO) terms: carotid artery dissection (HP:0012158), Pectus excavatum (HP:0000767), scoliosis (HP:0002650), agenesis of permanent teeth (HP:0006349), hearing impairment (HP:0000365), atrial septal defect (HP:0001631), inguinal hernia (HP:0000023) and abnormal finger morphology (HP:0001167). Given the patient’s vascular, skeletal, and connective-tissue-related features, a virtual panel analysis was performed using the WES data, including genes associated with heritable thoracic aortic disease (HTAD), arterial aneurysm/dissection, and syndromic connective tissue disorders. The analysis included, among others, FBN1, TGFBR1, TGFBR2, SMAD2, SMAD3, SMAD4, TGFB2, TGFB3, ACTA2, MYH11, MYLK, LOX, COL3A1, EFEMP2, FLNA, SKI, SLC2A10, and FBN2.

### 2.4. Case Presentation

The patient is an adult male, born in September 1990, who has been followed at the Adult Congenital Heart Disease (GUCH) clinic in Sweden because of complex congenital heart disease. At 8 months of age, he underwent cardiac surgery including direct closure of an ostium primum atrial septal defect (ASD), pulmonary valvulotomy, and repair of a cleft mitral valve. Additional congenital cardiovascular findings included a bicuspid aortic valve and mild dilatation of the ascending aorta.

In adulthood, the patient developed bilateral carotid artery dissections. The first dissection occurred in 2020 while he was in Thailand following severe coughing-associated neck extension/stretching. The second dissection occurred spontaneously in 2024 without any clear provoking factor. Extensive neurological investigations did not identify an alternative etiology. He was treated medically with acetylsalicylic acid and enalapril and has remained under continued follow-up by both cardiology and neurology services.

**Figure 2:**
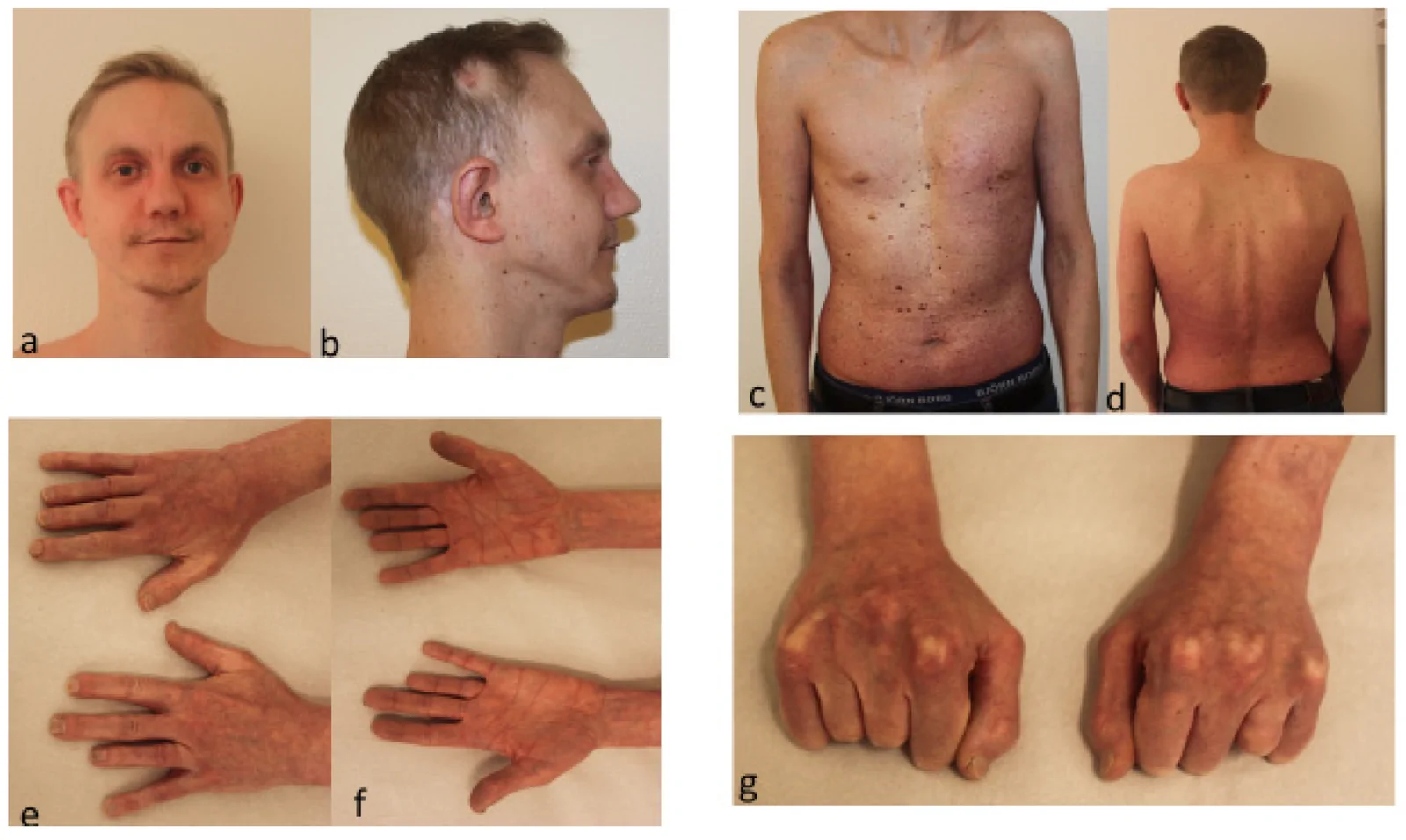
(**a**,**b**): Sparse and thin hair and eyebrow growth, thin lips, low-sitting ear and mandibular hypoplasia. (**c**,**d**): Scoliosis and diffuse vascular skin lesions with erythematous cutaneous changes. (**e**–**g**): Brachydactyly, broad thumbs, and short metacarpal bone.

A prominent feature of the patient’s presentation was a severe ectodermal dysplasia phenotype, with multisystem involvement affecting teeth, hair, skin, and nails (Figure 2). He had oligodontia with congenital absence of 11 permanent teeth, sparse and thin hair growth, hearing impairment, hyperopia, and abnormalities of the skin and nails consistent with ectodermal dysplasia. Dermatological evaluation documented diffuse vascular skin lesions with erythematous cutaneous changes involving the entire skin surface, plantar hyperkeratosis, dry skin of the hands, and painful fissures affecting the fingertips. His condition is followed regularly by a dermatologist because of chronic cutaneous manifestations related to the underlying syndrome, and he receives treatment with emollients and keratolytic therapy. The extensive ectodermal manifestations were considered highly characteristic of *PRKD1*-related telangiectasia–ectodermal dysplasia syndrome.

Additional syndromic manifestations included brachydactyly with abnormal finger morphology, scoliosis, mild intellectual disability, and learning difficulties.

Further medical history included asthma; recurrent pulmonary infections during childhood, including recurrent pneumonias; hypospadias and urethral abnormalities; infertility with reduced numbers of morphologically normal spermatozoa; chronic venous insufficiency with varicosities; recurrent erysipelas affecting the right leg; and cutaneous petechial lesions. He also has chronic parotitis and remains under follow-up by the ENT department. Due to concerns regarding lymphatic compromise and chronic venous insufficiency, he requires specialized compression stockings.

Family history revealed limited evidence of a clearly syndromic inherited disorder. His mother had suffered a cerebral hemorrhage at 54 years of age and reportedly also had hearing and visual impairment. One brother was reportedly healthy without apparent syndromic manifestations. There was otherwise no known family history of congenital heart disease, arterial dissections, or connective tissue disorders.

Genetic testing was first performed in 2025, as a unifying genetic diagnosis had not previously been considered despite the patient’s longstanding multisystem manifestations.

## 3. Results

SNP-microarray analysis: No copy number variants (CNVs) larger than 50 kb were identified that could explain the patient’s clinical phenotype.

Whole-exome sequencing: Exome sequencing identified a heterozygous, likely pathogenic *de novo* variant in *PRKD1* (NM_002742.3:c.1808G>A; p.(Arg603His)), consistent with PRKD1-related telangiectasia–ectodermal dysplasia–brachydactyly–cardiac anomaly syndrome. Virtual panel analysis of the WES data identified no additional pathogenic or likely pathogenic variants in genes associated with heritable thoracic aortic disease or connective tissue disorders that could provide an alternative genetic explanation for the patient’s vascular phenotype.

The variant affects a highly conserved residue within the kinase domain of the *PRKD1* protein and has previously been reported in association with *PRKD1*-related telangiectasia–ectodermal dysplasia–brachydactyly–cardiac anomaly syndrome (CHDED syndrome), an autosomal-dominant multisystem developmental disorder caused by gain-of-function variants in *PRKD1* [14]. Functional dysregulation of *PRKD1* has been implicated in cardiac morphogenesis, angiogenesis, endothelial signaling, inflammatory responses, vascular homeostasis, and cellular proliferation [7,13]. According to ACMG/AMP variant interpretation guidelines [19], the *PRKD1* c.1808G>A (p.Arg603His) variant is classified as likely pathogenic. This classification is supported by multiple lines of evidence, including absence or extremely low frequency in population databases (PM2), a phenotype highly specific for *PRKD1*-related disease (PP4), confirmed *de novo* occurrence with both parents testing negative (PS2_moderate), and supporting functional evidence consistent with a damaging effect on protein function (PS3_supporting). The PRKD1 c.1808G>A p.(Arg603His) variant is not in the gnomAD v4.1.0 or 8.3KJPN databases (allele frequency = 0) and is extremely rare in ExAC (allele frequency: 0.00001). Functional studies demonstrated constitutive, lipid-independent catalytic activity, supporting its gain-of-function effect. Taken together, these findings strongly support a causal role for the *PRKD1* variant in the patient’s multisystem phenotype.

The patient demonstrated substantial phenotypic overlap with previously reported *PRKD1*-associated disease [13,14], including congenital heart disease, oligodontia/agenesis of permanent teeth, sparse hair, hearing impairment, brachydactyly with abnormal finger morphology, learning difficulties, and cutaneous vascular abnormalities. In addition, the presence of bilateral carotid artery dissections and mild aortic dilatation may represent an expansion of the vascular phenotype associated with *PRKD1* dysregulation [1]. Given the biological role of *PRKD1* in angiogenesis and vascular integrity, a mechanistic association between the identified variant and arterial fragility is considered plausible. The coexistence of congenital cardiac disease, ectodermal dysplasia, vascular abnormalities, and connective tissue-related manifestations further supports a syndromic effect of altered *PRKD1* signaling.

TRIO-WES of proband and his parents demonstrated that neither parent carried the identified *PRKD1* variant, confirming that the c.1808G>A p.(Arg603His) alteration occurred *de novo* in the patient. This finding provides additional support for pathogenicity and is consistent with previous reports indicating that *PRKD1*-related CHDED syndrome frequently arises from *de novo* gain-of-function variants.

## 4. Discussion

*PRKD1*-related congenital heart defects and ectodermal dysplasia syndrome (CHDED) syndrome represents an exceptionally rare developmental disorder with only a limited number of cases reported worldwide (Table 1) [13,14]. The syndrome is primarily characterized by congenital cardiac malformations and ectodermal abnormalities, although the phenotypic spectrum continues to evolve as additional patients are identified. The present case significantly expands the clinical spectrum associated with *PRKD1* gain-of-function disease, particularly with respect to adult vascular manifestations. To date, only approximately eight patients with *PRKD1*-related telangiectasisa–ectodermal dysplasia–brachydactyly–cardiac anomaly syndrome have been reported worldwide [13,14,17,20]. The present patient demonstrates substantial phenotypic overlap with previously described cases, particularly with respect to congenital heart disease, ectodermal dysplasia, dental anomalies, brachydactyly, and neurodevelopmental involvement.

However, the occurrence of bilateral carotid artery dissections and aortic dilatation may represent a potential expansion of the vascular phenotype associated with *PRKD1* dysregulation. The circumstances surrounding the first dissection warrant consideration of both mechanical and intrinsic vascular factors. The first event followed severe coughing associated with neck extension/stretching and could plausibly have been precipitated by mechanical stress, as coughing, cervical hyperextension, and other forms of neck strain are recognized potential triggers for cervical artery dissection. Nevertheless, the subsequent spontaneous event, in the absence of an identifiable provoking factor, raises the possibility that the initial mechanical stress may have acted as a “second hit” in the setting of an underlying vascular susceptibility. During the adult clinical workup, systemic hypertension, fibromuscular dysplasia, and other acquired cardiovascular risk factors were evaluated, with no alternative etiology identified. Although a causal relationship between PRKD1 dysregulation and the vascular findings cannot be established from a single case, their occurrence may warrant consideration as part of the expanding vascular phenotype of this disorder. Further cases and functional studies, including patient-derived vascular models, are needed to determine whether altered PRKD1 signaling contributes to vascular fragility and arterial dissection.

Importantly, the potential mechanisms underlying the vascular phenotype should be considered in light of the distinct effects of PRKD1 loss-of-function and gain-of-function. Although PRKD1 loss-of-function mouse models demonstrate an essential role for PKD1 in cardiac development, these models do not directly recapitulate the heterozygous gain-of-function mechanism underlying CHDED. The largely normal cardiac development observed in heterozygous loss-of-function mice further suggests that reduced gene dosage and increased or dysregulated PKD1 activity may have distinct biological consequences. Notably, the c.1774G>C p.(Gly592Arg) variant reported by Alter et al. [14]. was associated with impaired substrate phosphorylation, suggesting a loss of kinase function, highlighting that different PRKD1 variants may have distinct functional effects. In contrast, gain-of-function variants may result in altered or increased downstream signaling, including MAPK/ERK and NFAT-related pathways, potentially affecting cellular differentiation, tissue morphogenesis, endothelial function, and vascular wall homeostasis. These mechanisms could provide a possible explanation for vascular abnormalities in individuals with PRKD1 gain-of-function variants, although functional studies are required to determine whether the p.(Arg603His) variant directly alters these pathways and contributes to vascular fragility.

Our patient exhibited many of the core features previously associated with *PRKD1*-related disease, including congenital heart defects, oligodontia, sparse hair, hearing impairment, skeletal abnormalities, and neurodevelopmental difficulties. The identified *PRKD1* c.1808G>A p.(Arg603His) variant has previously been described and experimentally associated with gain-of-function effects, supporting its pathogenic role in this patient’s phenotype [14]. To date, six *PRKD1* variants have been reported in eight patients with CHDED [20]. One variant (Figure 1 and Table 1) is located in the C1b domain, whereas the remaining five are in the kinase domain. Notably, in six patients, nucleotide substitutions occurred at two positions, 1774 and 1808, suggesting these sites may represent mutational hotspots in *PRKD1*.

Although the molecular diagnosis in our patient was established at 35 years of age, the clinical phenotype was evident from childhood, including congenital heart disease, ectodermal abnormalities, skeletal features, and neurodevelopmental difficulties. The later molecular diagnosis therefore reflects delayed genetic recognition rather than late disease onset. Notably, the previously reported PRKD1 variants were also identified as de novo, although functional studies have demonstrated differing effects on PKD1 activity. For example, the c.1774G>C p.(Gly592Arg) variant reported by Alter et al. [14] was associated with impaired substrate phosphorylation, suggesting a loss-of-function effect, in contrast to the gain-of-function mechanism established for the variant identified in our patient. This case highlights the importance of early genetic testing in patients with multisystem features suggestive of a syndromic disorder, which may facilitate earlier diagnosis, appropriate surveillance, and timely management.

The most notable and potentially novel finding in this case is the occurrence of bi-lateral carotid artery dissections. To our knowledge, spontaneous cervical arterial dissections have not been clearly reported previously in association with PRKD1-related CHDED syndrome. This observation raises the possibility that *PRKD1* dysregulation contributes not only to developmental abnormalities but also to vascular fragility and adult-onset arteriopathy. The biological plausibility of such an association is supported by the known functions of *PRKD1*. Protein kinase D1 participates in several signaling pathways involved in endothelial cell migration, angiogenesis, vascular remodeling, inflammatory signaling, oxidative stress responses, and smooth muscle cell regulation [7]. Dysregulation of these pathways could theoretically impair arterial wall integrity and predispose affected individuals to spontaneous dissections or aneurysmal disease. The presence of mild aortic dilatation in our patient may further support an underlying systemic vasculopathy.

Interestingly, the patient also demonstrated additional features suggestive of connective tissue or vascular involvement, including petechial skin lesions, chronic venous insufficiency with severe varicosities, and recurrent erysipelas associated with lymphatic compromise. Although these findings are nonspecific, their coexistence with arterial dissections may indicate a broader vascular phenotype than previously recognized in *PRKD1*-related disease.

Segregation analysis demonstrated that neither parent carried the identified *PRKD1* variant, confirming that the c.1808G>A p.(Arg603His) variant occurred *de novo* in the patient. Although the patient’s mother had a history of cerebral hemorrhage as well as hearing and visual impairment, no clinical evidence of *PRKD1*-related syndrome was identified in either parent. The *de novo* occurrence further strengthens the pathogenic role of the variant and is consistent with previous reports of *PRKD1*-related CHDED syndrome caused by *de novo* gain-of-function variants [14].

Another important aspect of this case is the adult presentation and long-term survival of a patient with *PRKD1*-related disease. Most previously reported individuals have been identified during childhood, often through investigations into congenital heart defects. This case illustrates that the phenotype may evolve significantly over time, with additional vascular and systemic manifestations emerging in adulthood.

The infertility observed in this patient may represent a previously unrecognized feature of PRKD1-related disease. Although PRKD1 signaling has been implicated in cellular differentiation and reproductive biology, the significance of the reduced sperm morphology in this single patient remains uncertain. Further clinical reports, particularly in male patients with PRKD1 gain-of-function variants, are needed to determine whether infertility or abnormal spermatogenesis is a recurrent feature of the disorder.

This report highlights the importance of considering genetic etiologies in patients presenting with unexplained arterial dissections in combination with congenital heart disease and multisystem developmental abnormalities. It also underscores the value of continued long-term follow-up of rare genetic syndromes, as adult phenotypes may differ substantially from pediatric presentations.

## 5. Conclusions

We report an adult patient with a likely pathogenic gain-of-function *PRKD1* variant presenting with congenital heart disease, ectodermal dysplasia, neurodevelopmental abnormalities, infertility, and bilateral carotid artery dissections. This case suggests a potential expansion of the known phenotypic spectrum of PRKD1-related CHDED syndrome and raises the possibility that vascular fragility and arteriopathy may represent previously underrecognized manifestations of the disorder.

The association between *PRKD1* dysregulation and arterial dissections is biologically plausible given the established role of *PRKD1* in angiogenesis, endothelial signaling, and vascular remodeling. However, causality cannot be established from a single case. Recognition of this potential vascular phenotype may have important implications for surveillance and long-term management of affected individuals. Further clinical and functional studies are needed to clarify the role of *PRKD1* in vascular integrity and to determine whether systematic vascular screening should be considered in patients with *PRKD1*-related syndromes.

## Figures and Tables

**Table 1:** Comparison of clinical and genetic variants and amino acid changes in PRKD1-related congenital heart defects and ectodermal dysplasia syndrome (CHDED). All PRKD1 variants listed in the table were reported as de novo. ASD: atrial septal defect, AVSD: atrioventricular septal defect, DD: developmental delay; ID: intellectual disability; ADHD: attention deficit hyperactivity disorder.

	Case 1	Case 2	Case 3	Case 4	Case 5	Case 6	Case 7	Case 8
	Present case	Sifrim et al. [17].	Sifrim et al. [17].	Sifrim et al. [17].	Alter et al. [14].	Alter et al. [14].	Alghaith et al. [20].	Leduc et al. [13].
*PRKD1* Variant	c.1808G>A	c.1774G>A	c.896T>G	c.1774G>A	c.1774G>C	c.1808G>A	c.1808G>T	c.2134G>A
PKD1 Protein	Arg603His	Gly592Arg	Leu299Trp	Gly592Arg	Gly592Arg	Arg603His	Arg603Leu	Val712Met
Protein domain	Kinase domain	Kinase domain	C1b	Kinase domain	Kinase domain	Kinase domain	Kinase domain	Kinase domain
Sex	M	M	M	M	M	F	M	F
Age at diagnosis (years)	35	6	7	5	18	19	2	14
Head and Neck	Oligodontia, sparse and thin hair growth, hearing impairment, hyperopia, erythematous skin, plantar hyperkeratosis, dry skin, recurrent parotitis, thin lip, low-sitting ear and mandibular hypoplasia	Microcephaly, Premature loss of primary teeth	Abnormal left eyebrows Widely spaced teeth, Prominent forehead, prominent nasal bridge, anteverted nares, full lips, Nystagmus	High anterior hairline, brachycephaly, depressed nasal bridge, frontal bossing, thin skin, pigmented naevi, Macrocephaly, Small, widely spaced teeth, Frontal bossing	Mild frontal bossing	Broad nasal bridge, mild maxillary and mandibular hypoplasia, high palate	Triangular face, bifid uvula, receding chin, micrognathia, long philtrum, maxillary and mandibular hypoplasia, depressed nasal bridge, prominent forehead and skull veins, low-set ears, prominent superior pinna, Bilateral papilledema, strabismus	Short philtrum, broad base of nose with short columella, protruding ears
Cardiovascular	ASD, Pulmonary, cleft mitral valve, bicuspid aortic valve, mild dilatation of ascending aorta, bilateral carotid artery dissection,	Pulmonary valve abnormality	AVSD	AVSD	Pulmonary stenosis	ASD, pulmonary stenosis	Partial AVSD, atrial stenosis, pulmonary stenosis, PAPVR	Partial AVSD, pulmonary stenosis, Right ventricle dysplasia
Abdomen	Feeding difficulties					Nephrocalcinosis	Altered gait, idiopathic intracranial hypertension, Nephrocalcinosis; medullary sponge kidney	Multicyclic renal dysplasia, left hypoplastic kidney, Gastroesophageal reflux, constipation
Skeletal	Scoliosis, Brachydactyly of hand,	Brachydactyly of hand, broad thumbs		Syndactyly of fingers and of II-III toes, deep plantar creases	Brachydactyly of hands and feet, cone-shaped epiphysis, partial II- III syndactyly of toe, Pectus excavatum	Brachydactyly of hands, Sternal deformity, Joint hypermobility	Transverse palmar creases, long toes, deep plantar creases	Brachydactyly of hands and feet, pes planus, lower limb asymmetry, cubitus valgus, capitate-trapezoid ankylosis Advanced bone age
Skin, nail and hair		Sparse scalp hair Premature loss of hair		Sparse scalp hair Small and widely dispersed	Sparse hair and eyebrows Tooth agenesis, Dry skin	Sparse hair, eyelashes and eyebrows	Sparse scalp hair Premature loss of primary teeth	Sparse hair and eyebrows Missing teeth

## Data Availability

The data presented in this study are not publicly available due to privacy and ethical considerations. The clinical information reported in this case report is derived from the patient’s medical records and is subject to restrictions to protect the patient’s privacy. Further information may be available from the corresponding author upon reasonable request and with appropriate permission, where applicable.
